# A Multi-View Three-Dimensional Scanning Method for a Dual-Arm Hand–Eye System with Global Calibration of Coded Marker Points

**DOI:** 10.3390/mi16070809

**Published:** 2025-07-13

**Authors:** Tenglong Zheng, Xiaoying Feng, Siyuan Wang, Haozhen Huang, Shoupeng Li

**Affiliations:** 1 School of Mechanical Engineering, Tiangong University, Tianjin 300387, China; 2120050108@tiangong.edu.cn; 2 College of Artificial Intelligence, Nankai University, Tianjin 300350, China; 3 Ocean College, Zhejiang University, Zhoushan 316021, China; wangsiyuan231600@163.com; 4 State Key Laboratory of Precision Measurement Technology and Instruments, Tianjin University, Tianjin 300072, China; huanghaozhen0913@126.com; 5 College of Electronic Information and Optical Engineering, Nankai University, Tianjin 300350, China; lishoupengnku@nankai.edu.cn

**Keywords:** three-dimensional vision system, dual robotic arms, hand–eye system, global calibration, multi-view measurement

## Abstract

To achieve robust and accurate collaborative 3D measurement under complex noise conditions, a global calibration method for dual-arm hand–eye systems and multi-view 3D imaging is proposed. A multi-view 3D scanning approach based on ICP (M3DHE-ICP) integrates a multi-frequency heterodyne coding phase solution with ICP optimization, effectively correcting stitching errors caused by robotic arm attitude drift. After correction, the average 3D imaging error is 0.082 mm, reduced by 0.330 mm. A global calibration method based on encoded marker points (GCM-DHE) is also introduced. By leveraging spatial geometry constraints and a dynamic tracking model of marker points, the transformation between multi-coordinate systems of the dual arms is robustly solved. This reduces the average imaging error to 0.100 mm, 0.456 mm lower than that of traditional circular calibration plate methods. In actual engineering measurements, the average error for scanning a vehicle’s front mudguard is 0.085 mm, with a standard deviation of 0.018 mm. These methods demonstrate significant value for intelligent manufacturing and multi-robot collaborative measurement.

## 1. Introduction

Driven by increasing market demand and the pressing need for technological innovation, the integration of robots with high-precision 3D vision has emerged as a crucial solution for flexible measurement challenges. In recent years, single-arm visual measurement systems have shown limitations, including low measurement speed, inefficiency, and an inability to perform full 3D inspection when target points fall outside the robotic workspace. To overcome these limitations, collaborative 3D measurement using dual robotic arms has gained increasing attention for its potential to achieve high-precision, efficient, and non-contact 3D inspection in complex measurement scenarios [1,2,3,4].

The widespread adoption of multi-camera systems and multi-robot measurement platforms in industrial manufacturing, healthcare, defense, and other sectors has attracted significant research attention to precise collaborative 3D measurement techniques. Hand–eye calibration is a critical prerequisite for collaborative 3D measurement in multi-robot systems. Its primary objective is to determine the precise geometric relationship between each robot end-effector and its corresponding vision sensor, thereby enabling coordinated measurement. Global calibration methods for multi-robot hand–eye systems have evolved into relatively mature frameworks encompassing theoretical modeling, visual inspection, data fusion, and optimization algorithms. However, they continue to face challenges in enhancing robustness, real-time performance, and accuracy under dynamic conditions [5,6,7]. To achieve global calibration in multi-robot vision systems, each robotic arm must acquire accurate and stable calibration data across diverse poses, followed by data processing and registration to establish coordinate transformations across multiple robot frames [8,9]. Wang et al. proposed an enhanced automatic TCP calibration method based on a laser displacement sensor and implemented on a cooperative robot with six degrees of freedom [10]. Daniele et al. proposed an attitude graph method to address the hand–eye calibration problem by leveraging the underlying graphic structure of the system, thus achieving global calibration of multiple robot hand–eye systems [11]. Wang proposed an algorithm based on bifurcation diagram analysis to investigate the system’s dynamic response under varying damping and stiffness parameters, effectively capturing stable symmetric orbits as well as periodic and chaotic motions [12]. Dominic et al. proposed a Kronecker–Product method to calibrate a dual-arm hand–eye system using a robot tracking tool by separating translation and rotation [13]. Erika et al. presented a multi-camera framework for collaborative robot vision servos, which enhanced the system’s robustness and efficiency [14]. Xu et al. developed a dual-robot calibration system using tooling components and established a mapping between the distance from reference points to the robot end-effectors and the base parameters, thereby accurately determining the relative pose between the two robots [15]. Davide et al. proposed a multi-camera hand–eye calibration method that optimizes the pose of each camera relative to the robot base and each other’s cameras [16]. Oliveira et al. proposed a novel calibration method for multi-sensor, multi-modal robot systems, utilizing the topological representation of coordinate systems to achieve global calibration across heterogeneous sensors [17]. Shahab et al. proposed a new control strategy for mobile manipulator systems (MMSs) that integrates image-based visual servoing (IBVS) to address operational limitations and safety constraints [18]. Zhou et al. proposed an optimization method based on the minimum spanning tree and graph for the calibration problem of multi-eye and multi-hand systems [19]. Wu et al. proposed a multi-sensor fusion calibration method, integrating hand–eye systems with IMUs for joint calibration of robot, camera, and IMU modules [20]. Wang et al. equated the multi-coordinate calibration problem of the dual-robot system to a matrix equation and employed a closed-loop method to obtain a rapid initial estimate for iterative refinement [21]. Wang et al. studied the visual tracking control problem in cooperative dual-robotic-arm systems and designed an adaptive algorithm to estimate the external parameters of the camera [22]. Duan et al. proposed a new novel hand–eye calibration method that combines spatial and out-of-plane constraints to iteratively optimize calibration parameters based on feature point relationships [23]. Yu et al. studied a hand–eye parameter estimation method based on 3D observation of a single marker, achieving global calibration for a large field-of-view 3D vision system [24]. Liu et al. proposed a vision-based synchronous calibration method that relies solely on marker position data, enhancing generalizability and reducing model dependency [25]. Tao et al. proposed a fast base frame calibration method based on the visual system, which can quickly and accurately calculate the relative pose between the hand–eye systems of a moving robotic arm [26].

At present, international researchers have conducted extensive studies on 3D measurement methods for robotic arm hand–eye systems, and several such visual systems have found applications in industrial metrology and intelligent manufacturing. However, under large field-of-view and high-noise conditions, collaborative 3D measurement using dual-arm hand–eye systems still encounters significant challenges in practical applications. Accurate motion pose estimation of the 3D vision system and proper normalization of the coordinate system are crucial for achieving high-precision collaborative 3D measurement with a dual-robotic-arm hand–eye system. The measurement field of view of the 3D vision sensor at the end of the robotic arm varies. In environments with background noise, field-of-view occlusions, and complex lighting, it is difficult to obtain real-time motion pose feedback from the 3D vision sensor. This limitation compromises the flexibility and accuracy required for global calibration, ultimately degrading the measurement performance of the dual-arm hand–eye system. Given the superior flexibility of encoded marker points, employing them for global calibration and multi-view 3D imaging of dual-arm hand–eye systems under non-overlapping fields of view and noisy conditions shows great potential. The main work and innovations of this paper are summarized as follows:

(1) M3DHE-ICP is proposed to significantly improve the consistency of multi-view point cloud stitching by introducing a refined correction process based on the ICP (iterative closest point) algorithm, which compensates for residual errors caused by calibration and 3D sensor measurement inaccuracies. The average Euclidean distance errors in fitting the center of a standard sphere using the dual-arm hand–eye system in multi-view 3D imaging were 0.082 mm and 0.066 mm, respectively;
(2) GCM-DHE is proposed to compute the global coordinates of encoded feature points through 3D reconstruction of a binocular motion structure. It further establishes the transformation relationships between the coordinate systems of two robotic arms by referencing encoded feature points and applying the Tsai–Lenz hand–eye calibration optimization algorithm. This method effectively addresses the challenges posed by non-overlapping fields of view and external noise. After calibration, the average error in multi-view 3D measurements using the dual-arm hand–eye system was 0.100 mm, demonstrating the effectiveness of the proposed global calibration approach;
(3) We conducted a global calibration experiment of the dual-arm hand–eye system under complex noise conditions and compared the results with other calibration methods to verify the robustness of the proposed approach. Subsequently, we carried out a practical 3D measurement experiment on an automobile front fender component using a binocular structured light vision system combined with dual robotic arms. The results showed an average measurement error of 0.085 mm and a standard deviation of 0.018 mm, further demonstrating the accuracy of the proposed method in practical measurement applications.

## 2. M3DHE-ICP

**Figure 1:**
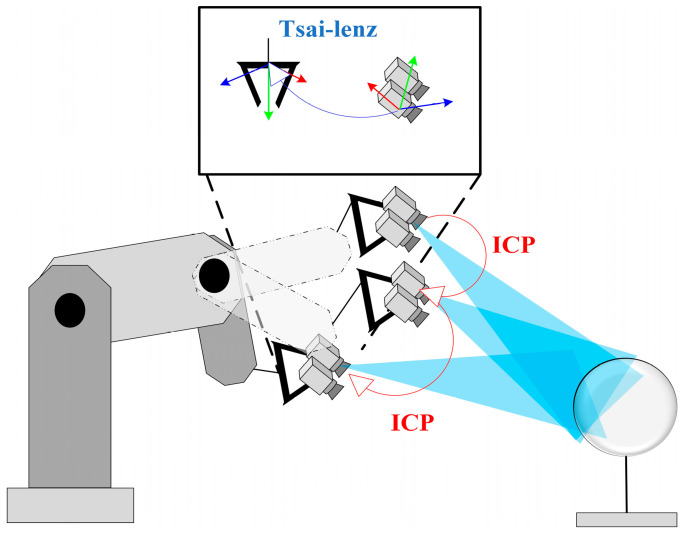
Schematic diagram of M3DHE-ICP.

The principle of M3DHE-ICP is shown in Figure 1. In the process of solving the hand–eye transformation matrix using the Tsai–Lenz algorithm, the relative poses between the robot coordinate system and the calibration plate coordinate system remain unchanged. Therefore, the transformation relationship between the robot coordinate system and the calibration plate coordinate system can be derived as shown in Equation (1).

(1) $$H_{{Cal}^{i}}^{{Base}^{i}}=H_{{Tool}^{i}}^{{Base}^{i}}{\cdot H}_{{Cam}^{i}}^{{Tool}^{i}}\cdot H_{{Cal}^{i}}^{{Cam}^{i}}$$

where $H_{{Cal}^{i}}^{{Base}^{i}}$ denotes the transformation matrix from the robot coordinate system of the ith robotic arm to the coordinate system of the calibration plate, $H_{{Tool}^{i}}^{{Base}^{i}}$ denotes the transformation matrix from the robot coordinate system of the ith robotic arm to the flange coordinate system thereof, $H_{{Cam}^{i}}^{{Tool}^{i}}$ denotes the transformation matrix from the flange coordinate system of the ith robotic arm to the camera coordinate system thereof, and $H_{{Cal}^{i}}^{{Cam}^{i}}$ denotes the transformation matrix from the camera coordinate system of the ith robotic arm to the coordinate system of the calibration plate.

By controlling the robotic arm’s vision system to acquire images of the calibration plate from multiple poses, transformation matrices can be obtained. Since the matrix $H_{{Cam}^{i}}^{{Tool}^{i}}$, representing the transformation from the flange coordinate system to the camera coordinate system, remains constant, Equation (2) can be derived from Equation (1).

(2) $${{(H_{{Tool}^{i}}^{{Base}^{i}}}_{j})}^{-1}\cdot {H_{{Tool}^{i}}^{{Base}^{i}}}_{k}\cdot H_{{Cam}^{i}}^{{Tool}^{i}}=H_{{Cam}^{i}}^{{Tool}^{i}}\cdot {H_{{Cal}^{i}}^{{Cam}^{i}}}_{j}\cdot {\left({H_{{Cal}^{i}}^{{Cam}^{i}}}_{k}\right)}^{-1}$$

where $H_{{Cam}^{i}}^{{Tool}^{i}}$ represents the transformation from the end-effector (flange) coordinate system to the camera coordinate system of the *i*th robotic arm. ${H_{{Tool}^{i}}^{{Base}^{i}}}_{j}$, ${H_{{Tool}^{i}}^{{Base}^{i}}}_{k}$, ${H_{{Cal}^{i}}^{{Cam}^{i}}}_{j}$, and ${H_{{Cal}^{i}}^{{Cam}^{i}}}_{k}$ denote the transformation matrices from the robot coordinate system of the robotic arm to the flange coordinate system and from the camera coordinate system to the coordinate system of the calibrated plate in the jth and kth positional states, respectively.

By converting the matrix equation in Equation (2) into the standard form AX=XB, the hand–eye transformation matrix for each robotic arm’s vision system can be obtained independently. The 3D point clouds from each robotic arm’s hand–eye system can be fused under multi-view conditions using the coordinate transformation equation shown in Equation (3).

(3) $$\Omega_{{Base}^{j}}^{P^{i}}=H_{{Base}^{j}}^{{Tool}_{i}^{j}}\left(R\right)*\left(H_{{Tool}^{j}}^{{cam}^{j}}\left(R\right)*\Omega_{{Cam}^{j}}^{P^{i}}+H_{{Tool}^{j}}^{{cam}^{j}}\left(T\right)\right)+H_{{Base}^{j}}^{{Tool}_{i}^{j}}\left(T\right)$$

where $\Omega_{{Base}^{j}}^{P^{i}}$ denotes the 3D point coordinates of the point cloud acquired by the jth robotic arm in the ith pose, expressed in its robot coordinate system. $\Omega_{{Cam}^{j}}^{P^{i}}$ denotes the 3D point coordinates of the point cloud acquired by the jth robotic arm in the ith pose, expressed in its camera coordinate system. A denotes the transformation matrix (including rotation and translation) from the robot flange coordinate system to the robot base coordinate system in the *j*th robotic arm’s hand–eye system at the jth pose. $H_{{Base}^{j}}^{{Tool}_{i}^{j}}$ denotes the transformation matrix (including rotation and translation) from the robot flange coordinate system to the robot base coordinate system in the jth robotic arm hand–eye system at the ith pose. $H_{{Tool}^{j}}^{{\mathrm{cam}}^{j}}\left(R\right)\;\mathrm{and}\;H_{{\mathrm{Tool}}^{j}}^{{cam}^{j}}\left(T\right)$ denote the rotation and translation matrices, respectively, from the camera coordinate system to the robot flange coordinate system in the jth robotic arm’s hand–eye system.

The cumulative motion error of the robotic arm hand–eye system and the correction of the collected 3D point cloud are determined using the optimization function shown in Equation (4).

(4) $${Loss}_{3d-3d}=arg\underset{\theta ,T}{\min}\sum_{i=1}^{N}{\left\|w_{i}\cdot (\Omega_{{Base}^{j}}^{i}-\left(R\left(\theta \right)\cdot \Omega_{{Base}^{j}}^{0}\right)+T\right\|}^{2}$$

where $R\left(\theta \right)$ and T denote the rotation and translation matrices obtained during the ICP iterative optimization process for aligning the 3D point cloud $\Omega_{{Base}^{j}}^{i}$ (from the ith pose) to the reference point cloud $\Omega_{{Base}^{j}}^{0}$ (from the 0th pose). $w_{i}$ denotes the weighting matrix that imposes a greater weight on the error in the Z-axis direction.

An iterative optimization model for multi-view 3D point cloud alignment in the robot coordinate system is constructed based on the Ceres solver, using the ICP correction loss function as shown in Equation (4). The hyperparameter settings of the iterative model are as follows: the error threshold in the Z-axis for transformed coordinates after stitching with the target point cloud is set to 0.1 to eliminate the influence of noise points; the weight coefficient $w_{i}$ is set to 20.0; the linear solver type in Ceres is configured as DENSE_QR; the output dimension of the residual is set to 3; and the maximum number of iterations is set to 200.

## 3. GCM-DHE

**Figure 2:**
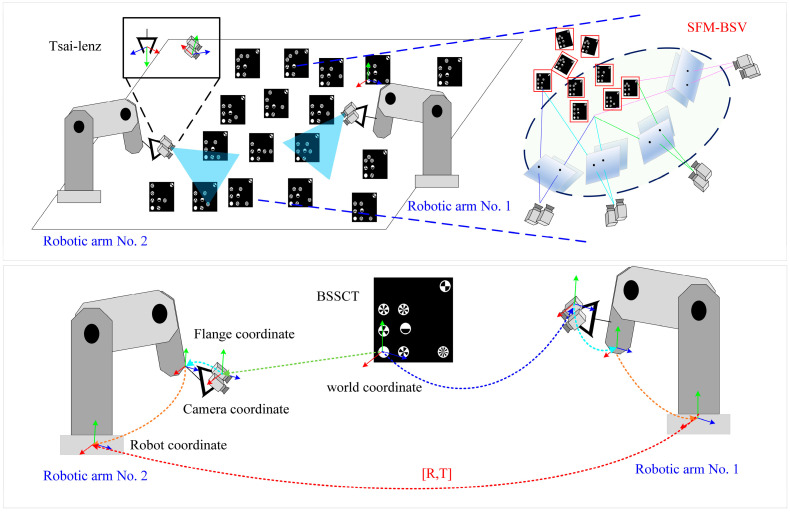
Multi-view 3D point cloud fusion method for dual-arm hand–eye system.

The principle of GCM-DHE is shown in Figure 2. The Tsai–Lenz algorithm is first applied to calibrate the hand–eye system of a single robotic arm. Then, the sub-pixel centers and 3D coordinates of the encoded feature points are localized using CSN-BSSCT [27], which generates a global 3D reference point cloud for the reconstruction of binocular motion via Structure from Motion by Binocular Stereo Vision (SFM-BSV). Next, SFM-BSV reconstructs the global 3D structure, synchronizes the calibration of external parameter matrices from multiple camera coordinate systems to the world coordinate system, and combines them with the camera-to-end-effector transformations obtained via the Tsai–Lenz algorithm to realize global calibration across. multiple robot coordinate systems.

**Figure 3:**
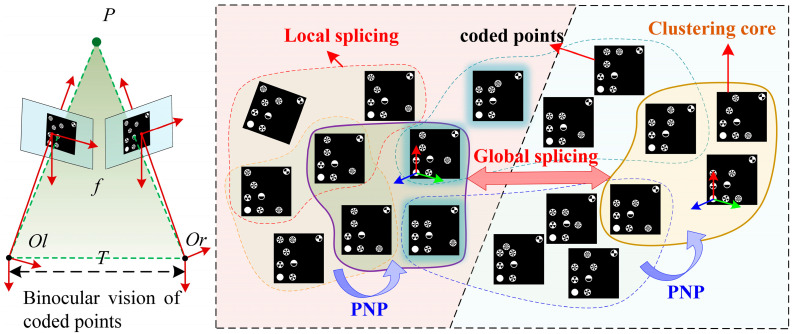
Schematic diagram of SFM-BSV.

The principle of SFM-BSV is illustrated in Figure 3. The left camera of the binocular vision system is selected as the reference coordinate system. The method constructs feature similarity relationships between multi-view encoded marker images in a graph structure and performs clustering of the encoded features. The feature similarity is quantified using a weight function, as defined in Equation (5), to characterize unordered image sets.

(5) $$\left\{\begin{array}{c} {∆}_{i}=\sum_{k=1}^{m_{i}}\sum_{l=1}^{m_{j}}\left(\sigma \left(f_{ik},f_{jl}\right)\right)\\ \sigma \left(f_{ik},f_{jl}\right)=\left\{\begin{array}{c} 1,if\left(f_{ik}=f_{jl}\right)\\ 0,if\left(f_{ik}\neq f_{jl}\right) \end{array}\right. \end{array}\right.$$

where ${∆}_{i}$ denotes edge weight in the sparse graph structure. $f_{ik}$ and $f_{jl}$ denote the features of the ith and jth-coded marker point in the image captured in the kth and  lth pose state, and $m_{i}$ and $m_{j}$ denote the number of features of the coded marker point in the image captured in the ith and jth pose state, respectively.

Based on the feature similarity equation shown in Equation (5), an adjacency-based clustering algorithm (Equation (6)) is designed to determine the center image of each unordered image cluster under different motion poses.

(6) $$\begin{array}{c} \forall \omega_{i}\in W,Adj\left(\omega_{i}\right)=\left\{\omega_{i}|{∆}_{ij}\geq \alpha \right\}\\ \underset{c_{k}\in C}{⋃}Adj\left(c_{k}\right)=W,C=arg\;\underset{C^{\prime }}{\min}C^{\prime } \end{array}$$

where $Adj\left(c_{k}\right)$ denotes the set of encoded feature images in the kth similarity clustering cluster, W denotes the set of unordered images of encoded marker points, $\omega_{i}$ denotes the feature image of the encoded marker points in the ith motion position state, ${∆}_{ij}$ denotes the feature similarity weight between the ith and jth images, $C^{\prime }$ denotes the set of images of clustering centers capable of covering all nodes, and C denotes the number of clustering centers minimized.

By constructing matched pairs of encoded feature points, spatial constraint equations in the binocular vision system can be derived as shown in Equation (7).

(7) $$\begin{array}{c} \begin{array}{c} \left(u_{l}ml_{31}-ml_{11}\right)x_{w}+\left(u_{l}ml_{32}-ml_{12}\right)y_{w}+\left(u_{l}ml_{33}-ml_{13}\right)z_{w}=ml_{14}-u_{l}ml_{34}\\ \left(v_{l}ml_{31}-ml_{21}\right)x_{w}+\left(v_{l}ml_{32}-ml_{22}\right)y_{w}+\left(v_{l}ml_{33}-ml_{23}\right)z_{w}=ml_{24}-v_{l}ml_{34} \end{array}\\ \begin{array}{c} \left(u_{r}mr_{31}-mr_{11}\right)x_{w}+\left(u_{r}mr_{32}-mr_{12}\right)y_{w}+\left(u_{r}mr_{33}-mr_{13}\right)z_{w}=mr_{14}-u_{r}mr_{34}\\ \left(v_{r}mr_{31}-mr_{21}\right)x_{w}+\left(v_{r}mr_{32}-mr_{22}\right)y_{w}+\left(v_{r}mr_{33}-mr_{23}\right)z_{w}=mr_{24}-v_{r}mr_{34} \end{array} \end{array}$$

where $ml_{ij}$ and $mr_{ij}$ denote the elements in the left and right camera projection matrices; $\left(u_{l},v_{l},1\right)$ and $\left(u_{r},v_{r},1\right)$ denote the chi-square coordinates of the encoded feature points in the left and right camera image coordinate system.

Based on the local partition clustering strategy defined in Equation (6), the center image of each local cluster of unordered images can be obtained. The coordinate system of the center images in multiple local clusters is used as the reference frame. The initial relative poses of other cluster nodes with respect to the cluster center are estimated using the PnP algorithm.

The feature similarity of each cluster is evaluated using the weight function from Equation (5). The two clusters with the highest similarity weights are selected as the initial matching module. An incremental global splicing model is then constructed using LM iterative optimization to reconstruct the 3D coordinates of encoded marker points in local clusters. The incremental splicing process transforms the 3D point clouds of all encoded marker clusters into the world coordinate system using Equation (8), thereby completing the global reconstruction.

(8) $${P_{global}={R^{global,i}}^{*}\cdot P}_{center}^{i}+{T^{global,i}}^{*},P_{center}^{i}\epsilon \left\{P_{center}^{1},P_{center}^{2},\ldots ,P_{center}^{n}\right\}$$

where $P_{global}$ denotes the global 3D point cloud of the encoded marker points in the large field of view; ${R^{global,i}}^{*}$ and ${T^{global,i}}^{*}$ denote the rotation and translation matrices of the ith cluster to the world coordinate system after iterative optimization by the LM algorithm, respectively; and $P_{center}^{i}$ denotes the 3D coordinates of the encoded marker points in the ith local cluster.

The spatial constraints among the image coordinate system, camera coordinate system, and world coordinate system in the dual-robotic-arm hand–eye system are established by encoding the position information of marker points in both the camera and image coordinate system, as shown in Equations (9) and (10).

(9) $$\left\{\begin{array}{c} {residual}_{ui}=\frac{u_{i}-\hat{u_{i}}}{f_{x}}\\ {residual}_{vi}=\frac{v_{i}-\hat{v_{i}}}{f_{y}}\\ E_{reproj}=\sum_{i}\left({residual}_{ui}^{2}+{residual}_{vi}^{2}\right) \end{array}\right.$$

where ${residual}_{ui}$ and ${residual}_{vi}$ denote the reprojection errors of the *i*th encoded marker point along the u and v directions in the image coordinate system. $u_{i}\;\mathrm{and}\;v_{i}$ are the observed subpixel coordinates, while $\hat{u_{i}}\;\mathrm{and}\;\hat{v_{i}}$ are the reprojected subpixel coordinates obtained using the transformation matrix $H_{{Cam}^{i}}^{world}$.

(10) $$\left\{\begin{array}{c} {error}_{eucl}^{i}=\sqrt{{\left(X_{w}^{i}-X_{c-w}^{i}\right)}^{2}+{\left(Y_{w}^{i}-Y_{c-w}^{i}\right)}^{2}+{\left(Z_{w}^{i}-Z_{c-w}^{i}\right)}^{2}}\\ Eeucl=\sum_{i}{\left({error}_{eucl}^{i}\right)}^{2} \end{array}\right.$$

where ${error}_{eucl}^{i}$ denotes the Euclidean distance error of the feature point, $P_{w}^{i}(X_{w}^{i},Y_{w}^{i},Z_{w}^{i})$ denotes the 3D coordinates of the encoded marker point in the world coordinate system, and $P_{c-w}^{i}(X_{c-w}^{i},Y_{c-w}^{i},Z_{c-w}^{i})$ denotes the 3D coordinates of the encoded marker point transformed from the camera coordinate system to the world coordinate system.

The 3D–3D Euclidean distance error is more strongly constrained to the absolute distance of the translation component, while the 3D–2D projection error is more sensitive to the direction of rotation and translation. Using the LM algorithm for iterative optimization of Equations (9) and (10), the position matrix between the corrected world coordinate system and the camera coordinate system can be solved for $H_{{Cam}^{i}}^{world}$. Using the transformation relationships among the coordinate systems of the dual robotic arms (as illustrated in Figure 2), Equation (2) is employed to obtain the position transformation matrices for each hand–eye system, specifically $H_{{Tool}^{1}}^{{cam}^{1}}\;\mathrm{and}\;H_{{Tool}^{2}}^{{cam}^{2}}$. Subsequently, the transformation matrix between the robot coordinate systems of arms No. 1 and No. 2 is computed using Equation (11).

(11) $$H_{{Base}^{2}}^{{Base}^{1}}=H_{{Tool}^{1}}^{{Base}^{1}}\cdot H_{{cam}^{1}}^{{Tool}^{1}}\cdot H_{world}^{{cam}^{1}}\cdot H_{{cam}^{2}}^{world}\cdot H_{{Tool}^{2}}^{{cam}^{2}}\cdot H_{{Base}^{2}}^{{Tool}^{2}}$$

where $H_{{Base}^{2}}^{{Base}^{1}}$ denotes a transformation relationship between the robot coordinate system of the robotic arm No. 1 to the robotic arm No. 2, $H_{{Cam}^{i}}^{{Tool}^{i}}$ denotes a transformation matrix from the flange coordinate system of the ith robotic arm to its camera coordinate system, $H_{{Base}^{i}}^{{Tool}^{i}}$ denotes a transformation matrix from the flange coordinate system of the ith robotic arm to its robot coordinate system, and $H_{{Cam}^{i}}^{world}$ denotes a transformation matrix from the world coordinate system to the camera coordinate system of the ith robotic arm.

## 4. Experiments

### 4.1. Multi-View 3D Imaging of the Hand–Eye System with M3DHE-ICP

**Figure 4:**
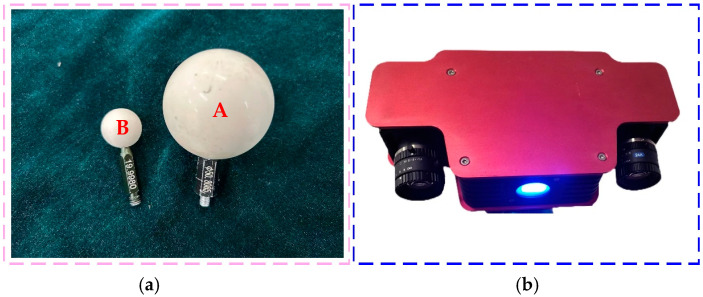
Equipment used in the 3D measurement experiment with the binocular structured light vision system: (**a**) standard sphere measurement target; (**b**) binocular structured light vision system and its components.

To evaluate the accuracy of the binocular structured light 3D vision system installed on the robotic arm, this study used a matte ceramic standard sphere (as shown in Figure 4a) as the measurement target. The binocular structured light vision system is shown in Figure 4b. As the perception module at the end of the robotic arm, the resolution of the camera is 1280 × 1024 pixels. The experiment adopted the improved three-wavelength six-step phase shift method to measure the three-dimensional shape of the standard sphere. The diameter of the matte ceramic standard ball A is 50.807 mm, and the diameter of the matte ceramic standard ball B is 19.998 mm.

**Figure A1:**
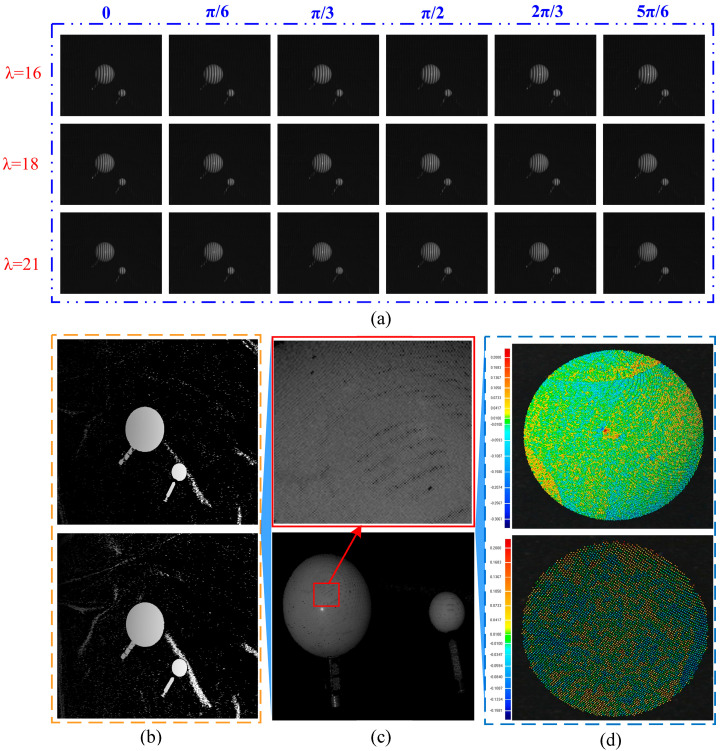
Three-dimensional measurement of a standard sphere using a binocular structured light vision system. (**a**) Deformation fringes of the standard sphere; (**b**) absolute phase map of the deformation fringes; (**c**) reconstructed 3D shape of the standard sphere; (**d**) spherical fitting error of the 3D measurement data.

The three-dimensional measurement results of a standard sphere using the binocular structured light vision system are shown in Figure A1. The No. 1 binocular structured light vision system achieved an average diameter fitting error of 0.022 mm for standard sphere A and 0.047 mm for standard sphere B. For the No. 2 system, the average diameter fitting errors were 0.018 mm for standard sphere A and 0.029 mm for standard sphere B. In summary, the average measurement error of the binocular structured light vision system in the dual-robotic-arm hand–eye system was 0.029 mm.

**Figure 5:**
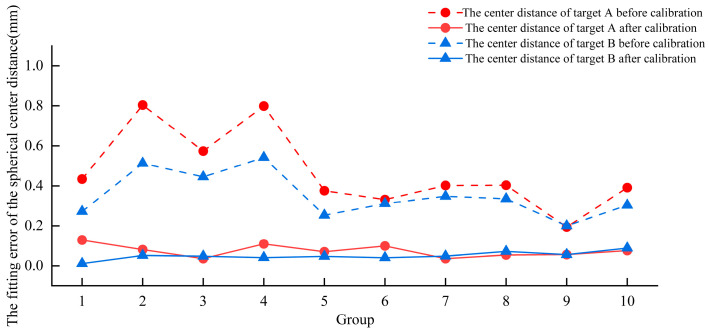
Euclidean distance errors of robotic arm hand–eye system No. 1 before and after correction for each set of spherical center coordinates in the multi-view fitting.

**Figure 6:**
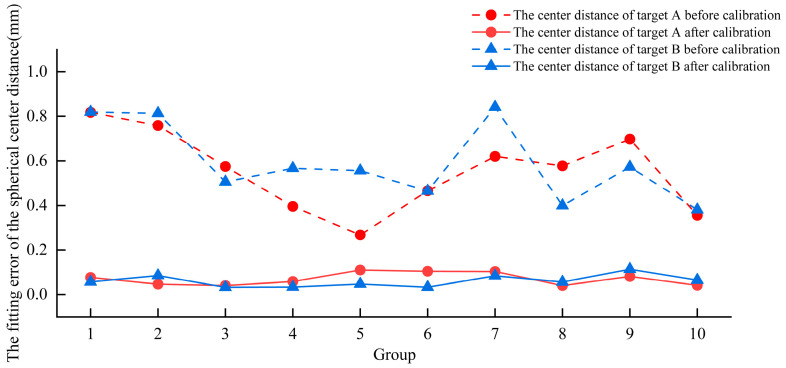
Euclidean distance errors of robotic arm hand–eye system No. 2 before and after correction for each set of spherical center coordinates in the multi-view fitting.

**Figure A2:**
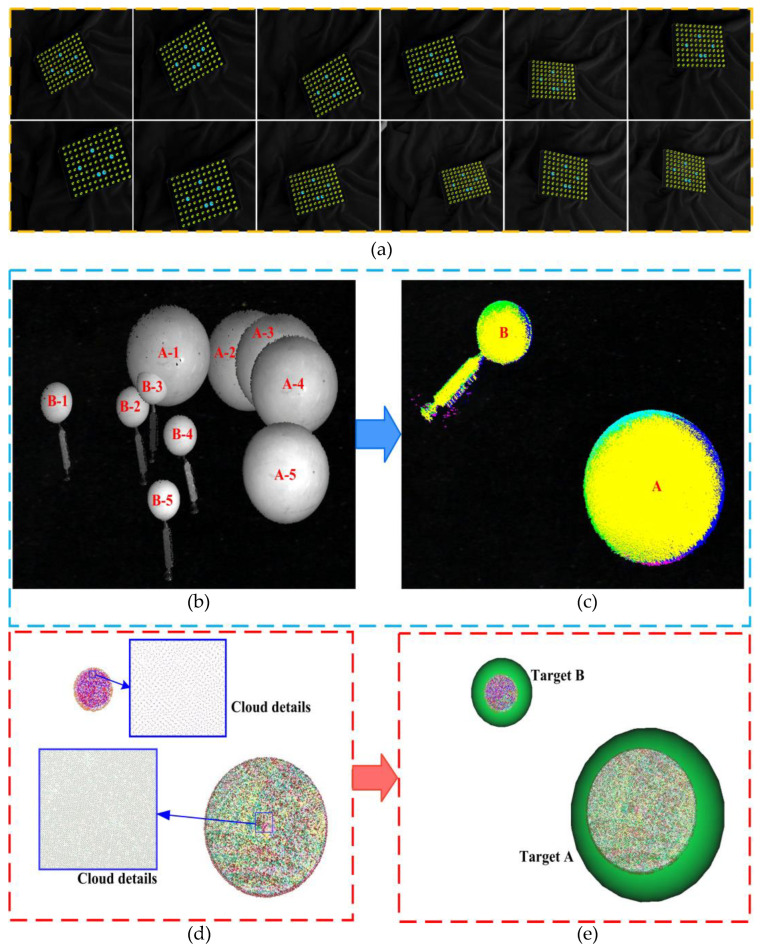
Optimal sphere coordinate fitting for multi-view 3D imaging of standard sphere by robotic-arm hand–eye system. (**a**) Calibration image of the hand–eye system; (**b**) 3D measurement results of the standard sphere using the binocular structured light vision system in multi-view; (**c**) multi-view 3D imaging of the standard sphere in the robotic coordinate system; (**d**) multi-view 3D point cloud data of the standard sphere after noise removal; (**e**) fitting results of the optimal sphere for the multi-view point cloud data.

Two robotic arms equipped with binocular structured light vision systems were used separately for multi-view collaborative 3D measurements. To evaluate the multi-view 3D imaging accuracy of each hand–eye system, an error analysis experiment was conducted on the best-fit center coordinates of the standard sphere across multiple views, as shown in Figure A2. The fitted spherical center coordinates of the first group of 3D data were used as a reference. Each subsequent group of spherical center coordinates was corrected and aligned in the robot coordinate system. The corresponding center distance errors before and after correction are shown in Figure 5 and Figure 6. As shown in Figure 5, before correction, the No. 1 robotic arm’s hand–eye system achieved an average Euclidean distance error of 0.471 mm for target A and 0.352 mm for target B. After correction, the average Euclidean distance errors were reduced to 0.076 mm for target A and 0.088 mm for target B. Compared with the pre-correction values, these represent reductions of 0.395 mm and 0.264 mm, respectively. As shown in Figure 6, prior to correction, the No. 2 robotic arm’s hand–eye system produced an average Euclidean distance error of 0.553 mm for target A and 0.592 mm for target B. After correction, the average Euclidean distance errors were 0.071 mm for target A and 0.061 mm for target B, representing reductions of 0.482 mm and 0.531 mm, respectively, compared to the uncorrected values. In summary, the average Euclidean distance errors of the spherical center fitting for multi-view 3D imaging were 0.082 mm and 0.066 mm for the hand–eye systems of robotic arms No. 1 and No. 2, respectively.

### 4.2. Three-Dimensional Scanning Imaging Tests of Multi-View Fusion for GCM-DHE

**Figure A3:**
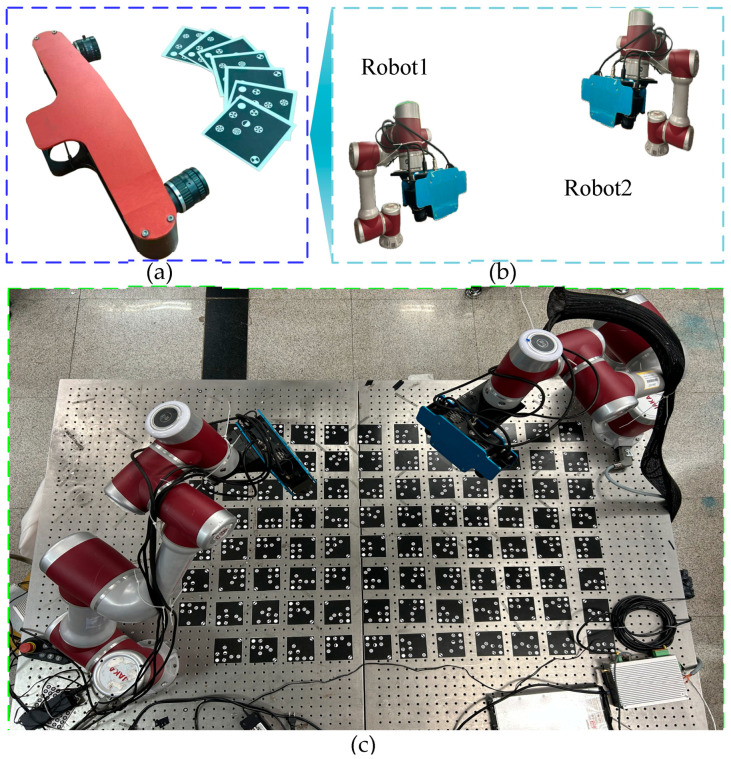
The hardware system for collaborative three-dimensional measurement of the hand–eye system of dual mechanical arms. (**a**) Dynamic three-dimensional tracking of BSSCT; (**b**) cooperative three-dimensional scanning of the hand–eye system of dual robotic arms; (**c**) global calibration of dual robotic arms for encoded marker points.

The hardware system for the collaborative three-dimensional measurement of the dual-robotic-arm hand–eye system is shown in Figure A3. Firstly, the BSSCT feature points in the non-overlapping field of view were collected through the binocular vision scanner as shown in Figure A3a, and the BSSCT feature detection was carried out by the CSN-BSSCT dynamic tracking module in GCM-DHE. The global reference three-dimensional point cloud was constructed through SFM-BSV. Then, through the dual-robotic-arm hand–eye system as shown in Figure A3b, the BSSCT feature images were collected as in the scene shown in Figure A3c. Finally, the dual-arm hand–eye system was calibrated through the Tsai–Lenz algorithm to achieve multi-view three-dimensional measurement of the dual-arm hand–eye system.

**Figure A4:**
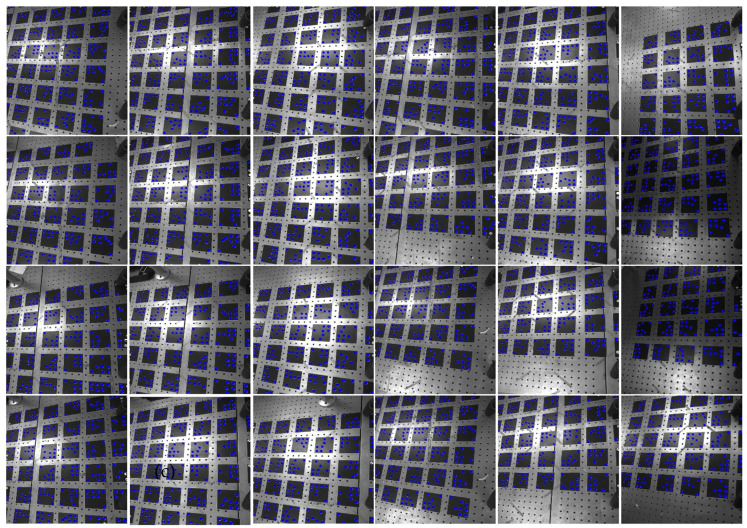
An example diagram of robustness detection of BSSCT feature points under complex noise conditions.

**Figure A5:**
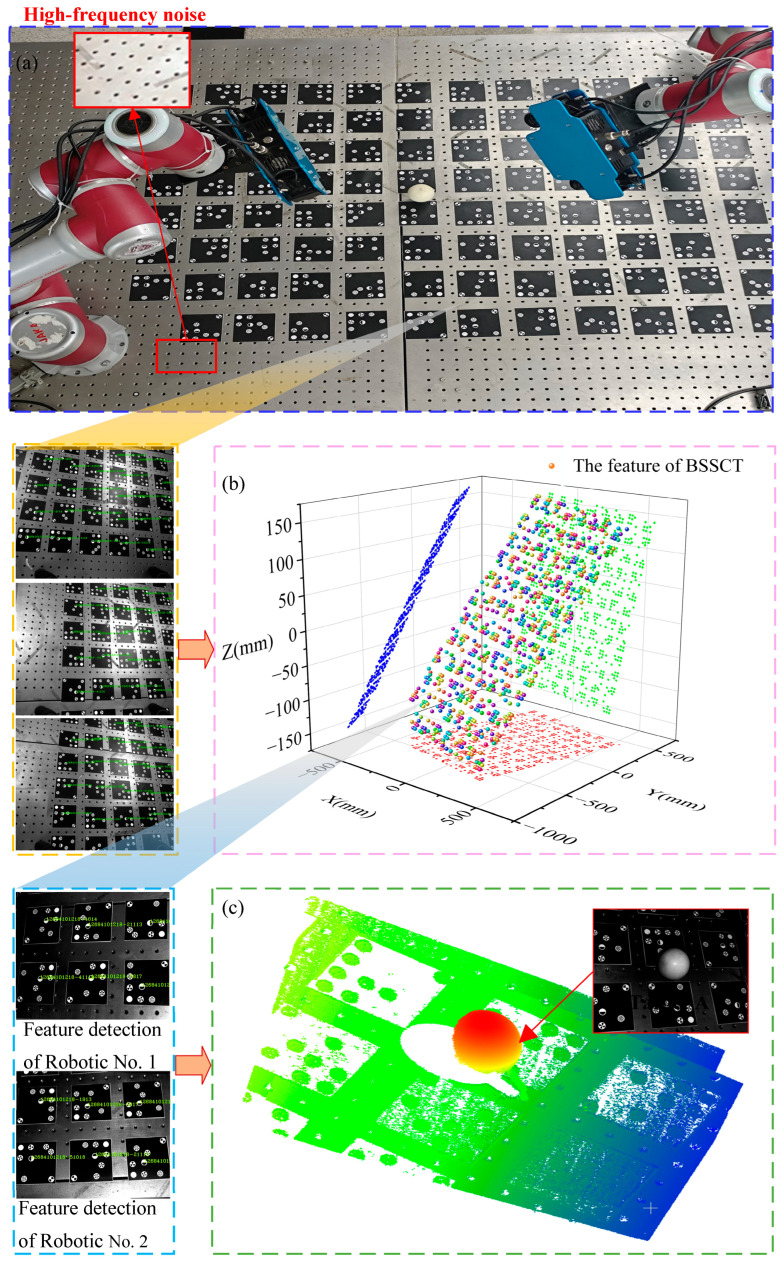
Global calibration results of GCM-DHE combined with Tsai–Lenz for a dual-robotic-arm hand–eye system. (**a**) Calibration environment under multi-noise interference; (**b**) construction of global reference 3D coordinates from BSSCT feature points using GCM-DHE; (**c**) multi-view 3D measurements of a standard sphere after global calibration.

GCM-DHE has the characteristics of a non-overlapping field of view and robustness. Supported by the NVIDIA GeForce RTX 3070 image processor, the BSSCT feature corner points extracted by GCM-DHE through CSN-BSSCT in complex scenes are shown in Figure A4, and the detection rate of each image was approximately 105.646 ms. The results of calibration of the dual-robotic-arm hand–eye system by GCM-DHE are shown in Figure A5. The calibration environment with multi-noise interference is shown in Figure A5a. Figure A5b displays the global reference 3D coordinate construction of coded marker points using SFM-BSV. The three-dimensional coordinates of the global point cloud of BSSCT constructed by the SFM-BSV module in GCM-DHE are shown in Table A1. Figure A5c presents the multi-view 3D measurement results of the standard sphere by the dual-robotic-arm hand–eye system after global calibration. Figure A5 shows that BSSCT can be detected rapidly under complex noise conditions through GCM-DHE, and the high-precision global calibration of the hand–eye system of the dual mechanical arms can be completed.

To verify the accuracy of the proposed method, feature images of the BSSCT were acquired using the binocular structured light vision system under three different motion poses of robotic arms No. 1 and No. 2, respectively, in order to compute the relative transformation between their coordinate systems. The 3D point cloud data acquired by the hand–eye system of robotic arm No. 1 from multiple viewpoints were transformed into the robot coordinate system of robotic arm No. 2. The average errors of the best-fit spherical center coordinates for these transformed point clouds are listed in Table 1. As shown in Table 1, the proposed global calibration algorithm enabled accurate multi-view 3D measurement of the standard sphere. The average fitting errors of the spherical center along the X-axis, Y-axis, and Z-axis were 0.044 mm, 0.028 mm, and 0.069 mm, respectively. Under three different motion pose groups, the Euclidean distance errors of the spherical centers from the multi-view 3D point clouds—transformed from the No. 1 to the No. 2 robot coordinate system—are given in Table 2. As shown in Table 2, the average Euclidean distance errors to the spherical center coordinates were 0.110 mm, 0.125 mm, and 0.065 mm for the first, second, and third motion pose states, respectively. The overall average error across all groups was approximately 0.100 mm.

**Figure A6:**
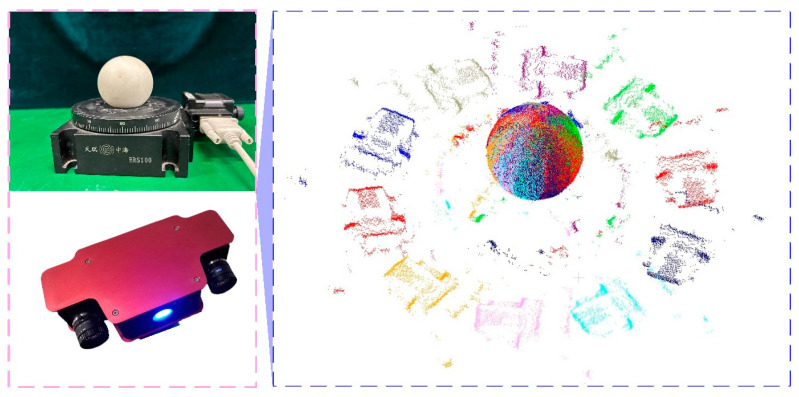
Results of multi-view 3D measurement of standard sphere by high-precision rotary table.

To compare the measurement accuracy of multi-view 3D imaging under different global calibration methods, a high-precision rotary table and a binocular structured light vision system (Figure A6) were used to perform 3D measurements of standard sphere A. The corresponding results are shown in Figure A6. In the overlapping field of view of the dual-robotic-arm hand–eye system, the two robot coordinate systems were globally calibrated using a circular calibration plate and the RANSAC-PnP algorithm for multi-view 3D measurements of target A. Across all motion poses of the robotic arm hand–eye system, the spherical centers obtained from multi-view 3D imaging of standard sphere A were fitted using different global calibration methods.

The accuracy comparison of multi-viewpoint three-dimensional imaging of target A by different global calibration methods is shown in Table 3. As shown in Table 3, compared with the rotary table scanning, handheld 3D scanner, and circular target method, the average spherical center fitting errors along the X-axis, Y-axis, and Z-axis of the method proposed in this paper were reduced by 0.064 mm, 0.074 mm, 0.004 mm, 0.072 mm, 0.048 mm, and 0.048 mm, respectively. Compared with the turntable method, the average Euclidean distance error of this method was reduced by 0.088 mm. Compared with the handheld 3D scanner method, the average Euclidean distance error of this method was reduced by 0.112 mm. Compared with the circular target method, the average Euclidean distance error was reduced by 0.456 mm. To sum up, the multi-view imaging method of the dual-robotic-arm hand–eye system proposed in our method has good global calibration robustness and high multi-view three-dimensional measurement accuracy under complex noise interference conditions. Since the binocular structured light system is a fixed-focus vision system, although GCM-DHE has strong robustness, when the depth of field of the camera exceeds the range, or the camera is in an out-of-focus state, it will lead to the loss of global calibration accuracy or calibration failure.

### 4.3. Actual Measurement Experiments of the Hand–Eye System with Dual Mechanical Arms

**Figure 7:**
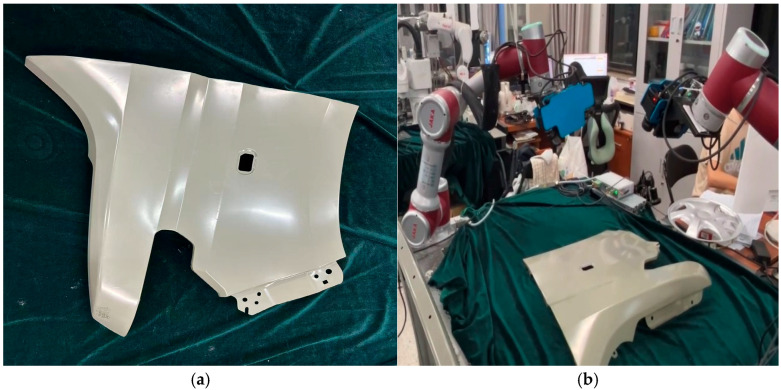
Three-dimensional measurement experiment of the car front fender using the dual-arm hand–eye system. (**a**) Car front fender; (**b**) dual-arm hand–eye system 3D measurement platform.

In order to evaluate the practical performance of the multi-view three-dimensional imaging method using the dual-arm hand–eye system, we measured the three-dimensional surface topography of the automotive part shown in Figure 7 through the dual-arm hand–eye system measurement platform shown in Figure A3.

**Figure 8:**
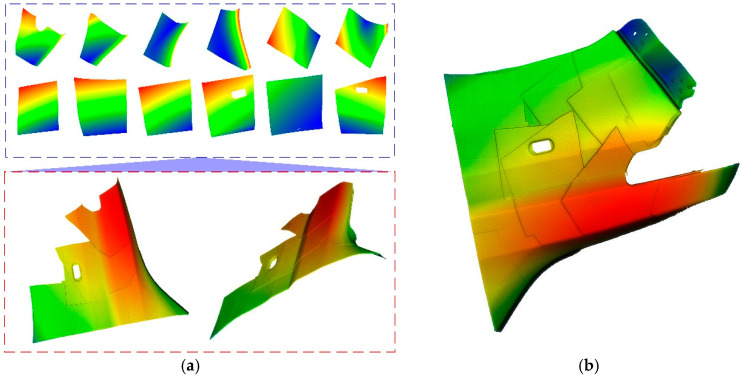
Multi-view 3D data acquisition and global point cloud. (**a**) Multi-view point cloud data collected by the dual-arm hand–eye system; (**b**) global 3D point cloud.

**Figure 9:**
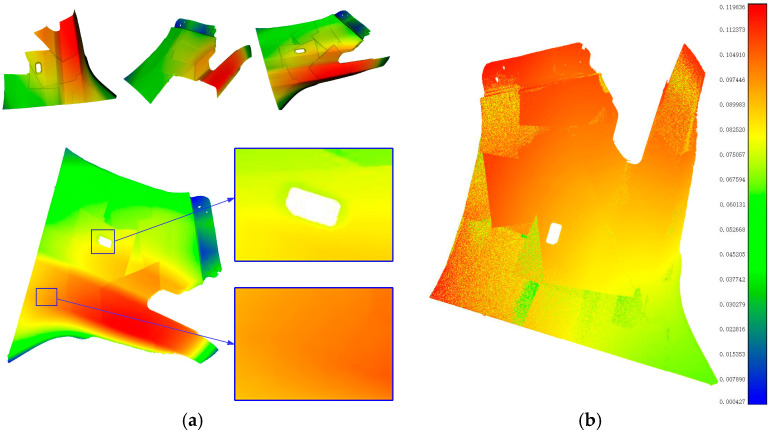
Accuracy analysis of 3D data acquisition for the car front fender by the dual-arm hand–eye system. (**a**) Global 3D point cloud corrected by ICP; (**b**) error mapping distribution map of the global 3D point cloud.

Figure 7a shows the front mudguard of the car to be tested, and Figure 7b shows the constructed dual-arm hand–eye system measurement platform. In the measurement experiment, the global calibration data of the hand–eye system of the dual mechanical arms are shown in Table A2. The three-dimensional point clouds collected from multiple perspectives are shown in Figure 8. Figure 8a shows the three-dimensional point clouds captured from multiple views and the global three-dimensional point clouds in each robot coordinate system, while Figure 8b presents the global three-dimensional point clouds in the world coordinate system. The error evaluation of the three-dimensional measurement results is shown in Figure 9. Figure 9a shows the global fusion point cloud optimized by ICP, and Figure 9b shows the average error distribution between the global three-dimensional point cloud and the standard model.

A coded marker-assisted global calibration method (GCM-DHE) for a dual-robotic-arm hand–eye system is proposed, combined with a multi-view 3D scanning method based on ICP correction (M3DHE-ICP). This approach effectively addresses the issues of inaccurate multi-coordinate system calibration and accumulated multi-view stitching errors under complex noise conditions. The GCM-DHE method establishes a robust global calibration relationship between the two robotic arms, and when combined with M3DHE-ICP, it significantly improves the accuracy of multi-view 3D measurements. In experiments, the spherical centroid fitting error of standard sphere imaging was controlled within 0.100 mm, and the average measurement error for the automobile front fender was 0.085 mm with a standard deviation of 0.018 mm, demonstrating the high accuracy and stability of the proposed method. The research results have important engineering application value and promotion prospects for multi-robot collaborative measurement and high-precision 3D reconstruction of complex workpieces in intelligent manufacturing environments.

## 5. Conclusions

A coded marker-assisted global calibration method (GCM-DHE) for a dual-robotic-arm hand–eye system is proposed, combined with a multi-view 3D scanning method based on ICP correction (M3DHE-ICP). This approach effectively addresses the problems of inaccurate multi-coordinate system calibration and accumulated multi-view stitching errors under complex noise conditions. The GCM-DHE method establishes a robust global calibration relationship between the two robotic arms. When combined with M3DHE-ICP, it significantly improves the accuracy of multi-view 3D measurements. In experiments, the spherical centroid fitting error for standard sphere imaging was controlled within 0.100 mm, verifying the high accuracy and stability of the proposed method. The research results have important engineering application value and promotion prospects for multi-robot collaborative measurement and high-precision 3D reconstruction of complex workpieces under an intelligent manufacturing environment. Based on this, in the subsequent research, the autonomous planning of the cooperative scanning path of the dual-robotic-arm hand–eye system will be the research goal.

## Figures and Tables

**Table 1:** Mean errors of best-fit spherical center coordinates for 3D measurement of the standard sphere using the dual-robotic-arm hand–eye system.

Mean Error of Fitted Spherocentric Coordinates (mm)/Group	ΔX	ΔY	ΔZ
1	0.022	0.036	0.040
2	0.073	0.029	0.062
3	0.036	0.018	0.104
All groups	0.044	0.028	0.069

**Table 2:** Euclidean distance fitting errors of spherical center coordinates for 3D measurements of the standard sphere using the dual-robotic-arm hand–eye system.

Mean Error of Fitted Spherical Center Distance (mm)/Group	3d Point Cloud of the 1st Motion Pose	3d Point Cloud of the 2nd Motion Pose	3d Point Cloud of the 3rd Motion Pose
1	0.080	0.066	0.044
2	0.097	0.147	0.092
3	0.153	0.163	0.059
All groups	0.110	0.125	0.065

**Table 3:** Comparison of multi-view 3D imaging accuracy using different global calibration methods.

Method/Fitting Mean Error (mm)	Rotary Table Scanning	Eva Handheld Scanner	Circular Calibration Target	Ours
ΔX	0.108	0.116	0.312	0.044
ΔY	0.102	0.076	0.281	0.028
ΔZ	0.065	0.117	0.270	0.069
Euclidean distance	0.188	0.212	0.556	0.100

## Data Availability

Data is contained within the article.

## Appendix A

**Table A1:** BSSCT reference three-dimensional coordinates for global calibration of the hand–eye system of the dual robotic arms.

Encode the Serial Number of the Feature Point	*X* (mm)	*Y* (mm)	*Z* (mm)
12684101218-11015-1	174.287	406.371	121.330
12684101218-11015-10	154.391	401.486	119.341
12684101218-11015-15	199.009	346.389	106.283
12684101218-11015-A	192.325	402.493	121.034
12684101218-11015-B	206.563	381.218	115.801
12684101218-11015-C	186.408	383.694	115.827
12684101218-11015-E	142.76	356.582	107.078
12684101218-11015-O	210.338	398.670	120.563
…			
12684101218-5817-17	169.153	−30.819	6.112
12684101218-5817-5	222.681	−7.008	14.042
12684101218-5817-8	195.773	−0.527	14.871
12684101218-5817-A	216.184	−32.987	7.053
12684101218-5817-B	193.861	−45.946	2.947
12684101218-5817-C	196.881	−26.520	8.204

**Table A2:** Global calibration data of the hand–eye system of the dual robotic arms.

	Variable	The Hand–Eye Matrix of Robotic Arm No. 1	The Hand–Eye Matrix of Robotic Arm No. 2	The Transformation Matrix of the Robot Coordinate System
Category				
Rotation		[0.133, 0.139, −1.568]	[−0.123, 0.231, 3.111]	[−7.582 × 10^−3^, −3.056 × 10^−3^, −0.746]
Translation		[−96.046, 65.053, 90.697]	[103.499, 103.559, 72.669]	[807.320, −1339.438, 20.040]
